# Supplementary material for: Determining mean and standard deviation of the strong gravity prior through simulations
Source: PLoS One. 2020 Aug 19;15(8):e0236732. doi: 10.1371/journal.pone.0236732 (PMC7446919; doi:10.1371/journal.pone.0236732)
Supplement: Attachment — Submitted filename: Response to Reviewers.pdf [file pone.0236732.s001.pdf]

Dear Prof. Dr. Baurès,

Thank you for the chance to submit a revised manuscript, and thank you particularly for your thorough handling of this manuscript.

In the following, you find our responses to both your and Reviewer 2’s comments.

Sincerely,

Björn Jörges

Editor:

As you will see in the reviews, I could possibly not receive more opposed reviews from the two reviewers. To help me getting a decision, I have contacted and been help by another academic editor that I wish to thank here, who has read and commented the reviews. We both agree that you have made substantial modifications that were asked by the second reviewer, in particular re-included S09 and excluded S10, which appears unnoticed by the second reviewer. It however appears that in referring to the previous article to describe the experimental design, you might have over-reacted. We feel that with the current article alone, it would be hard to a reader to understand the task. We should think that all readers might not have access to the previous publication, and hence the description of the task should stand on its own in the present manuscript.

We have extended the Methods section to reflect all relevant information.

Finally, the second reviewer still disagrees with the use of the mouse and the keyboard to collect temporal response. It seems to both me and the additional editor that with cautions on the software side, which you did, these two devices remain appropriate to collect these answers. However, I would recommend adding a few lines to present the reviewer's concern, with the citation, so that any reader can shape his own mind on the debate.

We have included Reviewer 2’s concern in the manuscript and have explained our strategy to mitigate delays and variability added by the input device.

Reviewer 2:

Given the abundance of errors in the previous version, I would have expected a cautious verification by the authors, but errors persist in the references of the figures (cf. lines 83, 91, 143, 145), in the typing errors (cf. line 67).

> We apologize. Some of these errors were introduced into the manuscript in the process of formatting the document for resubmission. We addressed these errors in this version.

I found it very disturbing to hide all the information about the methods (e.g., participants, task), especially because they hide important experimental questions raised in the previous expertise. This hides the nature of the participants.

> We had reduced the Methods section to minimize overlap with the published paper. As per your and the editor's request, we have re-included this information in this version of the manuscript.

In response to the authors, I urge them to distance themselves from colleagues who commonly integrate themselves as participants in their psychological experiences. It is absolutely necessary to avoid the authors being part of the population in psychological experiments in order to avoid introducing certain voluntary and involuntary biases in the behaviour of the experimenters. In the revised manuscript, it is not clear that the experimenters are part of the data analysed.

> We thank you for this suggestion. As stated in our previous response, we are highly in agreement with this concern and have removed the author from our analyses. The overall number of included participants remains the same (n = 9) because we re-included one participant we had previously excluded based on the editor's feedback.

In addition, the deletion of the method section hides the instruments used to monitor participant response. I continue to argue that the mouse and keyboard are not good tools for recording temporal responses despite the argument provided. Please read Plant & Turner, Behavior Research Methods, 2009. This makes the publication methodologically unacceptable.

> As per the editor's suggestion, we are making possible constraints as well as our mitigation efforts much clearer in the resubmitted version of the manuscript.

Finally, this deletion has led the authors to merge information regarding the exclusion of trials in the results section. I don't understand why the inclusion/exclusion of about 1% of the trials seems to be a necessity for the authors. What is behind all these efforts?

These trials include, for example, trials where the program froze, participants did not pay attention or other situations that led to extremely large recorded errors. These excluded trials do not reflect participant performance in any meaningful way.
